# Impact of a FTO gene risk variant on variables of energy metabolism in adults with obesity class 2 and 3

**DOI:** 10.1016/j.metop.2019.02.001

**Published:** 2019-02-19

**Authors:** Ann Kristin H. de Soysa, Marie Klevjer, Valdemar Grill, Ingrid Løvold Mostad

**Affiliations:** a Department of Clinical Nutrition and Speech-Language Therapy, Clinic of Clinical Services, St. Olavs Hospital - Trondheim University Hospital, Trondheim, Norway; b Department of Mathematical Sciences, Faculty of Information Technology and Electrical Engineering, Norwegian University of Science and Technology, Trondheim, Norway; c Department of Clinical and Molecular Medicine, Faculty of Medicine and Health Sciences, Norwegian University of Science and Technology, Trondheim, Norway

**Keywords:** Energy expenditure, Substrate oxidation, FTO, Obesity

## Abstract

**Purpose:**

The metabolic consequences of carrying a FTO obesity-promoting risk allele have not been fully elucidated and may be confounded by obesity *per se*. Against this background, we investigated the impact of FTO allele (SNP rs9939609) on fasting and postprandial energy expenditure and fasting substrate expenditure in a study population of uniformly and similarly obese individuals.

**Procedures:**

We studied a similar number of participants with BMI classes 2–3 (median BMI 42.8 kg/m^2^) who were either homozygote for the non-risk allele TT (n = 33, numbers increased by enrichment), heterozygote (AT) (n = 32), or homozygote for the risk allele AA (n = 35).

**Major findings:**

Basal metabolic rate and postprandial energy expenditure did not differ between FTO-groups. However, fasting respiratory quotient (RQ) was increased in those carrying ≥1 risk allele (p = 0.008), whereas postprandial RQ was not.

**Conclusion:**

In this study population, the FTO-risk allele associates with fasting reduced fat and increased carbohydrate oxidation.

## Highlights

• Fasting RQ associates with FTO risk allele in comparably obese subjects.
• The association remained after adjusting for gender effects.
• FTO risk not associated with meal-related energy utilization.

## 1 Introduction

The obesity-promoting effects of the fat mass and obesity-associated gene (FTO), risk allele is well studied, but the mechanisms behind effects are not fully elucidated [1]. One reason for remaining uncertainties might be the confounding influence of overweight *per se* on metabolic and behavioral parameters. To minimize such confounding we assessed energy expenditure (EE) and substrate utilization in FTO tested individuals with body mass index (BMI) obesity class 2–3. Inter-individual differences in food intake could potentially influence measurements [2,3]; therefore, we estimated each participants’ energy and macronutrient intake during the 24 h that preceded the overnight fast before measurements.

## 2 Subjects and methods

Patients newly referred to the hospital's obesity clinic were recruited to participate in an ongoing metabolic and genetic study. The first 50 participants who volunteered for genetic testing were selected randomly. The following 50 patients were included through a selection procedure blinded to investigators and participants. The selection procedure aimed to ensure three equal-sized groups of risk alleles (AA, AT, and TT). The Regional Ethics Committee (Trondheim, Norway) approved the study. Participants gave written informed consent and we conducted the study according to the Declaration of Helsinki.

One hundred patients aged ≥20y who met inclusion-criteria of BMI≥40 kg/m^2^ or ≥35 kg/m^2^ with comorbidities except type-2 diabetes-mellitus and not being pregnant/lactating participated.

We extracted deoxyribonucleic acid (DNA) from peripheral blood leukocytes from ethylenediaminetetra-acetic acid (EDTA) whole blood using the Gentra Purgene Blood Kit (QIAGEN Science, Germantown, USA), and performed genotyping of rs9939609 FTO using 7900HT Fast Real-Time polymerase chain reaction (PCR) System and predesigned TaqMan single nucleotide polymorphism (SNP) genotyping assays (Life Technologies/Thermo Fisher Scientific, Waltham, USA) specified for the SNP. A positive and a negative control were included on each sample tray [4].

Participants arrived in the morning after fasting for 10 h. Height was measured to the nearest 0.1 cm and weight (in underwear) to the nearest 0.1 kg. We collected blood samples preprandially, and analyzed serum glucose using BIOSEN C_line, sport (EKF-diagnostic GmbH, Barleben, Germany). Participants then consumed, in ≤15 min, a breakfast of whole-grain bread, butter, cheese, jam, orange juice, and milk or sweetened drinking yoghurt [2512 kJ (600 kcal), 17%, 35%, and 48% of energy from protein, fat, and carbohydrate, respectively] [5].

Energy expenditure, oxygen consumption (VO_2_), carbon dioxide production (VCO_2_), and respiratory quotient (RQ) were measured pre- and postprandially by indirect calorimetry using a ventilated hood system (Vmax Encore 29, CareFusion, Hoechberg, Germany), with recordings every 30sec for 15min per session. One session pertained to the fasting, and five sessions to the 2.5 h postprandial stage. There was a 30min interval between the start of each session. Steady state in indirect calorimetry is reportedly obtained when the coefficient of variance of VO_2_ and VCO_2_ is ≤ 10% [3]. Accordingly, we chose only data points between 6.5min and 14min for analysis for each session.

We calculated fasting carbohydrate and fat oxidation using each participant's basal metabolic rate (BMR) (kcal), RQ value, and a revised table of non-protein RQ [6].

Trained dietitians conducted 24 h dietary recalls on the day preceding the metabolic testing. We estimated portion sizes using Norkost-3 picture booklet,^1^ and calculated nutrient intake with KBS version 7.3.^2^ We retrieved self-reported information on smoking habits from the participants’ electronic medical journal.

On a separate day, participants underwent body composition-measurements (head, trunk, and legs) by dual energy x-ray absorptiometry (Holigic, Inc., Apex Software, Bedford, USA).

Using Stata 15 software [7] we performed univariate analyses on gender differences and differences between those carrying ≥1 risk variant of rs9939609 FTO gene polymorphism (AA + AT alleles) and those carrying the non-risk variant (TT allele). Since a few of our observations appeared to be outliers we used the more robust Wilcoxon-Mann-Whitney's nonparametric test to compare groups.^3^ We then tested all the variables in regression models robust to heteroscedasticity and outliers with the risk FTO risk-groups as the independent variable. We also controlled for gender in selected models due to significant gender differences on some outcomes.

## 3 Results

### 3.1 Characteristics of the study population

Thirty-three participants (27 women) were homozygote for the FTO non-risk allele TT, 32 (20 women) were heterozygote for the risk allele AT, and 35 (23 women) were homozygote for the risk allele AA (Table 1). Expectedly, men had higher stature, weight, LBM, energy-, fat- and protein-intake than women (Table 1). Risk and no-risk participants were similar in age, height, weight, BMI, and lean body mass (LBM) (Table 2). There were no significant differences in energy or macronutrient intake, or smoking habits between the risk- and no-risk groups (Table 2).

**Table 1:** Study population (n = 100, 70% women): Characteristics, energy and substrate oxidation.

Variable	Median	5^th^ percentile	95^th^ percentile	Mean	SD^a^	p-value^b^
Age (year)	42	25	62	41.9	11.5	0.644
Height (cm)	170	158	186.5	171.1	8.7	<0.001
Weight (kg)	122.4	95.5	162.8	126.6	21.3	<0.001
Body Mass Index (kg/m^2^)	42.8	35.7	50.4	43.1	5.2	0.152
Lean body mass^c^ (kg)	60.1	46.7	88.1	64.2	12.7	<0.001
Fat mass^d^ (kg)	44.6	29.0	63.8	5.6	0.7	0.191
Fasting serum glucose (mmol/L)	5.5	4.75	6.66	2132	816	0.037
Energy^e^ (kcal)	2098	1094	3835	91.85	33.35	0.004
Protein^e^ (g)	87.5	46.2	152.3	217.1	106.4	0.314
Carbohydrate^e^ (g)	199	73	440.2	94	44.02	0.054
Fat^e^ (g)	87.4	32.7	186.2	1,3	6.59	0.841
Alcohol^e^ (g)	0	0	4.4	1,9	4.41	0.921
Cigarettes per day	0	0	12.5	41.9	11.5	0.644
BMR^f^ (kcal)	1609	1231	2243	1651	91	<0.001
Postprandial total EE^g^ (kcal)	1808	1434	2342	1827	284	<0.001
Meal induced EE above BMR^f^ (kcal)	184	−49	355	177	109	0.093
RQ fasting^f^	0.850	0.76	0.97	0.856	0.061	0.346
RQ postprandial^g^	0.878	0.803	0.946	0.878	0.038	0.738
CHO oxidation fasting^f^ (mg/min)	144.9	54.3	304.2	159.2	69.8	0.001
Fat oxidation fasting^f^ (mg/min)	54.0	11.1	90.1	54.5	23.9	0.071

a SD: standard deviation.

b Wilcoxon-Mann-Whitney's test, men vs women. Significant if p ≤ 0.002 (Bonferroni correction).

c Lean body mass is defined as lean mass of trunk + left and right lower extremities.

d Fat mass is defined as fat mass of trunk + left and right lower extremities.

e Calculated from 24 h dietary recall preceding meal test.

f n = 98.

g n = 99.

**Table 2:** FTO non-risk allele vs risk alleles: Characteristics, energy and substrate oxidation.

Variable	No-risk allele^a^ n = 33 (82% women)					Risk alleles^b^ n = 67 (64% women)					p-value^d^
	Median	5^th^ percentile	95^th^ percentile	Mean	SD^c^	Median	5^th^ percentile	95^th^ percentile	Mean	SD^c^	
Age (year)	39	25	61	39.4	10.9	44	25	63	43.1	11.7	0.127
Height (cm)	169	156	188	170.8	9	170	158	184	171.2	8.6	0.826
Weight (kg)	119.2	94.2	171.1	125.4	23.7	126.6	95.7	159	127.2	20.1	0.519
Body Mass Index (kg/m^2^)	41.7	34.6	53.9	42.8	5.6	42.9	36	49.9	43.2	5.0	0.714
Lean body mass^e^ (kg)	59	46.7	88.4	62.76	13	61.8	47.8	85.9	64.9	12.60	0.386
Fat mass^f^ (kg)	46.3	28.5	62.5	46.7	10.38	44.2	31.1	63.8	45.3	10.224	0.468
Fasting serum glucose (mmol/L)	5.4	4.6	6.6	5.4	0.5	5.6	4.9	6.7	5.7	0.80	0.012^j^
Energy^g^ (kcal)	1973	1088	3246	1994	683	2099	1099	4211	2200	871	0.310
Protein^g^ (g)	78.3	45.3	146	85.3	29.4	90.6	47.1	152.6	95.1	34.90	0.164
Carbohydrate^g^ (g)	191.8	72	351.1	202.7	92.4	203.9	83.8	471.3	224.4	112.65	0.512
Fat^g^ (g)	89.8	32.7	160.1	88.8	43.77	87.1	27.7	187.8	96.6	44.24	0.335
Alcohol^g^ (g)	0	0	0	0.7	3.76	0	0	7.4	1.6	7.62	0.388
Cigarettes per day	0	0	12.5	2	4.27	0	0	12.5	1.9	4.51	0.127
BMR^h^(kcal)	1600	1252	2295	1628	283	1627	1231	2152	1663	297	0.478
Postprandial total EE^i^ (kcal)	1808	1434	2350	1818	293	1800	1441	2309	1832	281	0.630
Meal induced EE above BMR^h^ (kcal)	186	−11	355	191	106	182	−52	345	170	110	0.548
RQ fasting^h^	0.840	0.74	0.89	0.832	0.044	0.860	0.78	0.97	0.868	0.065	0.008^k^
RQ postprandial^i^	0.878	0.79	0.918	0.874	0.037	0.879	0.816	0.946	0.88	0.039	0.755
CHO oxidation fasting^h^ (mg/min)	136.2	33.0	242.6	135.1	57.2	150.4	83.2	315.1	171.4	72.7	0.021^l^
Fat oxidation fasting^h^ (mg/min)	61.1	37.3	87.5	62.4	16.6	50.5	9.8	90.1	50.5	26	0.011^m^

a TT (homozygote non-risk allele).

b AA + AT (homozygote + heterozygote risk alleles).

c SD: standard deviation.

d Wilcoxon-Mann-Whitney's test, no-risk allele (TT) vs. risk alleles (AA and AT). Significant if p ≤ 0.002 (Bonferroni correction).

e Lean body mass is defined as lean mass of trunk + left and right lower extremities.

f Fat mass is defined as fat mass of trunk + left and right lower extremities.

g Calculated from 24 h dietary recall preceding the meal test.

h n = 98.

i n = 99.

j The risk effect is positive and significant even after controlling for gender (p = 0.020, Coef. 0.294).

k The risk effect is positive and significant even after controlling for gender (p = 0.002, Coef. 0.035).

l The risk effect is positive and significant even after controlling for gender (p = 0.029, Coef. 26.407).

m The risk effect is negative and significant even after controlling for gender (p = 0.001, Coef. −13.998).

### 3.2 Fasting stage

Participants in the risk-group displayed slightly higher fasting serum glucose than no-risk participants (Table 2). BMR was similar for the risk- and the no-risk groups. As expected, men's BMR was higher than women's (median 1910 kcal vs 1523 kcal).

Fasting RQ was higher in the risk-than in the no-risk group (median 0.860 vs 0.840, p = 0.008). This difference remained significant also after excluding two subjects with glucose >6.99 mmol/l (p = 0.008) and after controlling for gender (p = 0,002, Coef.0.035) (Table 2).

### 3.3 Postprandial stage

Postprandial EE (total, and meal induced EE above BMR) did not differ between risk- and no-risk groups. In the whole study population, postprandial EE was lower in women (median 2090 kcal in men vs 1690 kcal in women, p < 0.001). Postprandial RQ did not differ between risk- and no-risk groups, and not between genders for the sample as a whole.

## 4 Discussion

Numerous studies have reported on the FTO risk allele's impact on obesity. Even so, our study brings out novel findings. Our study population is rather unique as it compares the impact of the FTO gene on metabolic parameters in subjects who are uniformly obese, thus obviating a confounding influence of obesity *per se*. Our main and novel finding is a higher fasting RQ in subjects carrying the FTO risk allele, particularly in women. The finding appears robust to the influence of potential dietary confounders because the dietary intake between the two groups was similar. Dietary intake on the day prior to testing is unlikely to have influenced our results on fasting RQ [2,8]. Risk-allele individuals displayed somewhat higher fasting blood glucose levels than no-risk-allele individuals. The difference (mean and median) was, however, minor, and glucose levels were within normal range. Fasting RQ, thus, should not have been affected by a glucose factor. The impact of FTO-risk on fasting RQ is not just statistically significant, but its substantive effect is also quite large. Carrying ≥1 risk-allele on average increases fasting RQ by 58% of a standard deviation of fasting RQ (Coef. 0.035/SD fasting RQ).

Further, the differences in RQ translate into a meaningful difference between groups with lower fat- and higher carbohydrate oxidation among risk-allele individuals. Specifically, in the fasted state risk individuals appear on average, to burn roughly 6.7 kcal/h less fat than no-risk individuals. A higher RQ and lower fat oxidation could be in line with a reported lower frequency of browning adipocytes associated with the risk allele [9]. Hence, browning adipocytes would likely be more metabolically active than white adipocytes and consequently oxidize more fat [10]. Browning adipocytes would likely be less metabolically active postprandial situation; this, we speculate, could explain the absence of an allele association with postprandial RQ. Further research is needed to elucidate a possible link between body weight and the herein reported differences in RQ.

A large Dutch study failed to detect differences in RQ based on FTO alleles [10]. The discrepancy with our findings could relate to designs: The Dutch study was an epidemiologic one, encompassing all levels of BMI. Our study was restricted to markedly obese people and designed to match the degree of obesity in subjects with or without the FTO risk allele. Furthermore, we anticipate that the enrichment of homozygotes in our study population would increase the possibilities of detecting differences in metabolic parameters between groups. However, we acknowledge that one cannot extrapolate the differences in RQ that we find here to lesser degrees of adiposity without further research. In other respects, our data agree with those of others that do not find effects of the FTO risk haplotypes on BMR or postprandial EE [1]. We also find expected metabolic differences between men and women. In our view, these concurring data adds evidence to the validity of our novel findings.

There are limitations to our study. Given that our subjects were all patients who volunteered, our sample might suffer some selection bias. Further studies in population-based individuals with BMI ≥35 are thus needed to confirm our results.

In conclusion, in a study population of uniformly and similarly obese individuals we find that a FTO risk allele associates with reduced fat and increased carbohydrate oxidation.

## Author contributions

ILM and VG conceived the study. ILM recruited participants. AKDS and MK analyzed EE data. AKDS, ILM and VG wrote the manuscript. All authors reviewed and edited the manuscript and have nothing to disclose. There is no conflict of interest.

## Declaration of interest

None.

## List of abbreviations

BMI: body mass index
BMR: basal metabolic rate
CHO: carbohydrate
cm: centimeter
DNA: deoxyribonucleic acid
EDTA: ethylenediaminetetra-acetic acid
EE: energy expenditure
FTO: fat mass and obesity-associated gene; alpha-ketoglutarate dependent dioxygenase
h: hour, hours
kg: kilogram
kcal: kilocalorie
kJ: kilojoule
LBM: lean body mass
min: minute, minutes
PCR: polymerase chain reaction
RQ: respiratory quotient
SNP: single nucleotide polymorphism
VCO_2_: rate of carbon dioxide production
VO_2_: rate of oxygen consumption

## Footnotes

1 *Norkost 3: Bildehefte med porsjonsstørrelser* developed at the Department of Nutrition, University of Oslo in cooperation with the Norwegian Food Safety Authority and the Norwegian Directorate of Health.

2 *KBS version 7.3, 2017* is an in-house data program based on the official Norwegian food composition table, developed at the University of Oslo.

3 Using the standard student t-test gave similar results.
